# Metformin Protects Against Radiation-Induced Pneumonitis and Fibrosis and Attenuates Upregulation of Dual Oxidase Genes Expression

**DOI:** 10.15171/apb.2018.078

**Published:** 2018-11-29

**Authors:** Rasoul Azmoonfar, Peyman Amini, Hana Saffar, Saeed Rezapoor, Elahe Motevaseli, Mohsen Cheki, Rasoul Yahyapour, Bagher farhood, Farzad Nouruzi, Ehsan Khodamoradi, Dheyauldeen Shabeeb, Ahmed Eleojo Musa, Masoud Najafi

**Affiliations:** ^1^Radiology and Nuclear Medicine Department, School of Paramedical Sciences, Kermanshah University of Medical Science, Kermanshah, Iran.; ^2^Department of Radiology, Faculty of Paramedical, Tehran University of Medical Sciences, Tehran, Iran.; ^3^Clinical and Anatomical Pathologist at Tehran University of Medical Science, Imam Khomeini Hospital Complex, Tehran, Iran.; ^4^Department of Molecular Medicine, School of Advanced Technologies in Medicine, Tehran University of Medical Sciences, Tehran, Iran.; ^5^Department of Radiologic Technology, Faculty of Paramedicine, Ahvaz Jundishapur University of Medical Sciences, Ahvaz, Iran.; ^6^School of Medicine, Jiroft University of Medical Sciences, Jiroft, Iran.; ^7^Departments of Medical Physics and Radiology, Faculty of Paramedical Sciences, Kashan University of Medical Sciences, Kashan, Iran.; ^8^Department of Medical Radiation Engineering, Science and Research Branch, Islamic Azad University, Tehran, Iran.; ^9^Department of Medical Physics and Biomedical Engineering, Faculty of Medicine, Tehran University of Medical Sciences (International Campus), Tehran, Iran.; ^10^Department of Physiology, College of Medicine, University of Misan, Misan, Iraq.; ^11^Research Center for Molecular and Cellular Imaging, Tehran University of Medical Sciences (International Campus), Tehran, Iran.

**Keywords:** Radiation, Metformin, Inflammation, Fibrosis, DUOX1, DUOX2

## Abstract

***Purpose:*** Lung tissue is one of the most sensitive organs to ionizing radiation (IR). Early and late side effects of exposure to IR can limit the radiation doses delivered to tumors that are within or adjacent to this organ. Pneumonitis and fibrosis are the main side effects of radiotherapy for this organ. IL-4 and IL-13 have a key role in the development of pneumonitis and fibrosis. Metformin is a potent anti-fibrosis and redox modulatory agent that has shown radioprotective effects. In this study, we aimed to evaluate possible upregulation of these cytokines and subsequent cascades such as IL4-R1, IL-13R1, Dual oxidase 1 (DUOX1) and DUOX2. In addition, we examined the potential protective effect of metformin in these cytokines and genes, as well as histopathological changes in rat’s lung tissues.

***Methods:*** 20 rats were divided into 4 groups: control; metformin treated; radiation + metformin; and radiation. Irradiation was performed with a ^60^Co source delivering 15 Gray (Gy) to the chest area. After 10 weeks, rats were sacrificed and their lung tissues were removed for histopathological, real-time PCR and ELISA assays.

***Results:*** Irradiation of lung was associated with an increase in IL-4 cytokine level, as well as the expression of IL-4 receptor-a1 (IL4ra1) and DUOX2 genes. However, there was no change in the level of IL-13 and its downstream gene including IL-13 receptor-a2 (IL13ra2). Moreover, histopathological evaluations showed significant infiltration of lymphocytes and macrophages, fibrosis, as well as vascular and alveolar damages. Treatment with metformin caused suppression of upregulated genes and IL-4 cytokine level, associated with amelioration of pathological changes.

***Conclusion:*** Results of this study showed remarkable pathological damages, an increase in the levels of IL-4, IL4Ra1 and Duox2, while that of IL-13 decreased. Treatment with metformin showed ability to attenuate upregulation of IL-4–DUOX2 pathway and other pathological damages to the lung after exposure to a high dose of IR.

## Introduction

Nowadays, cancer treatment using ionizing radiation (IR), also known as radiotherapy, is one of the most common modalities. The aim of radiotherapy is to eradicate all tumor cells with lowest possible damage to the surrounding normal tissues.^1^ However, radiation therapy is associated with some side-effects. Early and late side effects of exposure to high doses of radiotherapy may limit the radiation doses delivered.^2^ Also, a high dose of radiation may affect the long‐term quality of life of cancer patients.^3^ lung inflammation and fibrosis is one of the most important late effects of radiotherapy which may appear months or years after treatment. These side effects are usually associated with damage to vascular structures, infiltration of immune system cells such as macrophages, lymphocytes and mast cells in addition to oxidative damage and tissue remodeling.^4^ Radiation-induced fibrosis develops through long term changes in some biological elements and processes, such as cytokines and growth factors, inflammation, fibroblast differentiation etc.^5^

Several experiments have proposed TGF-β, IL-4 and IL-13 signaling pathways as the most important pathways involved in radiation-induced fibrosis.^6^ TGF-β through stimulation of Smad pathway, promotes fibroblast differentiation.^7^ IL-4 stimulates both IL-13 and TGF-β. which further amplifies the process of fibrosis. Furthermore, IL-4 and IL-13 stimulate production of free radicals, a long time after exposure, resulting in chronic oxidative stress.^8^ It has been shown that IL-4 has a two-fold effect on promotion of fibrosis.^9^ Upregulation of Dual oxidase 1 (DUOX1) and DUOX2 by these cytokines play a key role in chronic ROS production, inflammation and fibrosis.^10^ Moreover, it has been shown that IL-4 plays a key role in the maintenance of macrophages in lung tissues following exposure to radiation. On the other hand, macrophages are the main source of IL-4 production.^11^ It has been suggested that chronic infiltration of macrophages through production of IL-4, plays a central role in the development of pneumonitis and lung fibrosis.^12^ Suppressing these genes can ameliorate various side effects induced by IR.

So far, various agents such as herbal compounds, antioxidants etc., have been examined for preventing radiation-induced pneumonitis and lung fibrosis.^13,14^ Metformin has shown some radioprotective and antioxidant effects.^15^ Moreover, this drug has shown ability to ameliorate fibrosis induced by various toxic agents such as chemotherapy drugs.^16,17^ Metformin has been shown to possess antioxidant effect via direct neutralization of ROS and stimulating antioxidant enzymes.^18^ However, a major effect of metformin is its stimulatory effect on DNA repair pathways through upregulation of AMP-activated protein kinase (AMPK).^19^ AMPK is able to stimulate BER and HR pathways of DNA repair, leading to amelioration of clastogenic agents such as IR.^20,21^ Metformin also has a potent inhibitory effect on mitochondrial electron transfer chain 1 (ETC1), leading to attenuation of superoxide production in oxidative stress conditions. In this study, we examined the protective effect of metformin (in a non-toxic dose) on development of radiation-induced inflammation and fibrosis associated with other pathological changes such as vascular damage. In addition, we evaluated its effects on changes in the levels of pro-fibrotic cytokines such as IL-4 and IL-13 and their downstream genes, including IL-4Ra1, IL-13Ra2, DUOX1 and DUOX2.

## Materials and Methods

### Animal preparation 

20 adult male Wistar rats were purchased from Razi institute, Tehran University of Medical Sciences, Tehran, Iran. Rats weighing 200 ± 20 g were housed in accordance to the principles outlined in “The Guide for The Care and Use of Laboratory Animals” prepared by Kermanshah University of Medical Sciences. All rats were kept under controlled conditions, including humidity (60 ± 5%), temperature (25 ± 2°C), as well as 12h light and dark cycle.

### Metformin treatment

Metformin powder was prepared by Tehran Chemie Pharmaceutical Company, Tehran, Iran. It was dissolved in distilled water at a concentration of 20mg/ml. Oral administration of the resulting solution was done 4 and 5 consecutive days before and after irradiation, respectively. On the day of irradiation, metformin was administered 30 minutes before irradiation. To obtain a non-toxic drug dose as well as optimum radioprotective effect, a 100 mg/kg dose of metformin was selected based on previous studies.^22^

### Irradiation of animals 

Before irradiation, animals were anesthetized using intraperitoneal injection of ketamine 10% at a dose of 80 mg/kg and xylazine 2% at a dose of 5 mg/kg. The rats were irradiated on the thoracic region with a ^60^Co source of gamma rays at a dose rate of 109 cGy/min. A single dose of 15 Gy was selected for inducing lung injury based on previous study by Ghosh et al.^23^

### Experimental design

Rats were divided into four groups. Group 1 (control): 5 rats served as controls without any intervention. Group 2 (metformin): 5 rats were treated with metformin for 10 consecutive days. Group 3 (radiation): 5 rats received 15 Gy gamma rays to their chest area. Group 4 (metformin + radiation): 5 rats were treated with metformin for 4 and 5 consecutive days before and after irradiation, respectively. All animals sacrificed 10 weeks after irradiation. Their lung tissues were removed after chest surgery. The right lung tissues were frozen immediately at -80°C for ELISA and real-time analysis, while the left parts were fixed in 10% neutral buffered formalin for histopathological assay.

### Real-time PCR

Lung tissues were homogenated and total RNA was extracted. The concentration of total RNA was evaluated by a nanodrop. The extracted RNAs were transcribed to cDNA using cDNA Synthesis Kit (Gene All, South Korea). Afterwards, the expression of IL-4R, IL-13R, DUOX1 and DUOX2 were quantified using Corbett PCR system (USA) and their amplifications were performed with master mix green (Ampliqon). Expression of these mentioned genes was quantified relative to the reference gene and normalized to phosphoglucomutase 1 (PGM1) as the housekeeping gene. The genes’ primer sequences were designed using Gene Runner software and BLAST from NCBI. The primer sequences of IL-4R, IL-13R, DUOX1, DUOX2 and PGM1 are shown in Table 1.

**Table 1:** The primer sequences of genes which were used for real-time PCR.

**Gene**	**Forward sequence**	**Reverse sequence**
**IL-4R1**	GAGTGAGTGGAGTCCCAGCATC	GCTGAAGTAACAGGTCAGGC
**IL-13Ra2**	TCGTGTTAGCGGATGGGGAT	GCCTGGAAGCCTGGATCTCTA
**DUOX1**	AAGAAAGGAAGCATCAACACCC	ACCAGGGCAGTCAGGAAGAT
**DUOX2**	AGTCTCATTCCTCACCCGGA	GTAACACACACGATGTGGCG
**PGM1**	CATGATTCTGGGCAAGCACG	GCCAGTTGGGGTCTCATACAAA

### Enzyme-Linked Immunosorbent Assay (ELISA)

Lung tissues were homogenated by a homogenizer device. The levels of IL-4 and IL-13 in the lung homogenates were detected by Rat IL-4 and IL-13 ELISA kits (Zelbio, Germany) according to the manufacturer’s instructions.

### Histopathological evaluation

After sacrificing the rats, their left lungs were removed and fixed in formalin, and then embedded in paraffin. Sections of the removed lungs were cut into 5 µm, stained with hematoxylin and eosin (H and E) and Masson's trichrome (MTC). All pathological analyses were performed at the pathology unit of Imam Khomeini Hospital, Tehran, Iran. The blinded pathological study was performed with a light microscope using a semi-quantitative scoring system for the detection of histological parameters including fibrosis, edema, vascular damage and immune cells infiltration.

### Statistical analysis

The results were presented as mean ± standard deviation and *P*<0.05 was considered statistically significant. Data were analyzed using SPSS16 for Windows, Chicago, USA. Results of real-time PCR were analyzed by t-test while histopathological and ELISA results were analyzed using one-way ANOVA with post*-*hoc Tukey’s HSD.

## Results and Discussion

Lung is one of the most radiosensitive but late responding organ to radiotherapy. During radiotherapy for lung cancer or other tumors adjacent to the lung, late effects such as pneumonitis and fibrosis may threaten the life of patients. Moreover, exposing the lungs to radioactive particles after a radiation disaster or during a non-uniform whole body irradiation may cause death due to pneumonitis or fibrosis, months or years after exposure.^24^ In recent years, several studies have been conducted to illustrate the molecular mechanisms of radiation injury to the lung. Also, some studies have proposed several agents which inhibit inflammatory and profibrotic pathways to protect or mitigate lung pneumonitis and fibrosis.^25-28^ Evidences have shown that inflammatory and profibrotic cytokines play a key role in chronic consequences of exposure to IR in lung tissue.^29^ Amongst several cytokines, IL-1, IL-4, IL-6, IL-13, TNF-α and TGF-β are the most effective cytokines in promoting late effects of radiation injury in various organs such as the lung.^24,30^ IL-4 and IL-13 are able to induce continuous oxidative stress which mediate chronic inflammation and fibrosis. Studies have shown that IL-4, through its receptor IL-4R1, upregulates the expression of both DUOX1 and DUOX2, while IL-13 can upregulate IL13R and DUOX1.^31,32^ This may continue for a long time after exposure to IR, leading to disruption of normal function of tissues and increased risk of carcinogenesis.^30,33^ In addition, IL-4 has a key role in infiltration of macrophages in the lung and subsequent consequences such as promotion of fibrosis and inflammation.^34^

### Signs of radiation sickness 

The results of the survival rates of control, metformin treated, irradiation and irradiation plus metformin, evaluated at the end of the 10^th^ week after local-thorax irradiation showed that rats which received metformin had healthy signs similar to the control group. In addition, those rats which were treated before and after irradiation, did not show any sign of radiation sickness. However, rats which received gamma rays without metformin had signs of reduced weight (possibly because of problems in food and water intake), epilation and ruffling of their hairs.

### Real-time PCR

**Figure 1:**
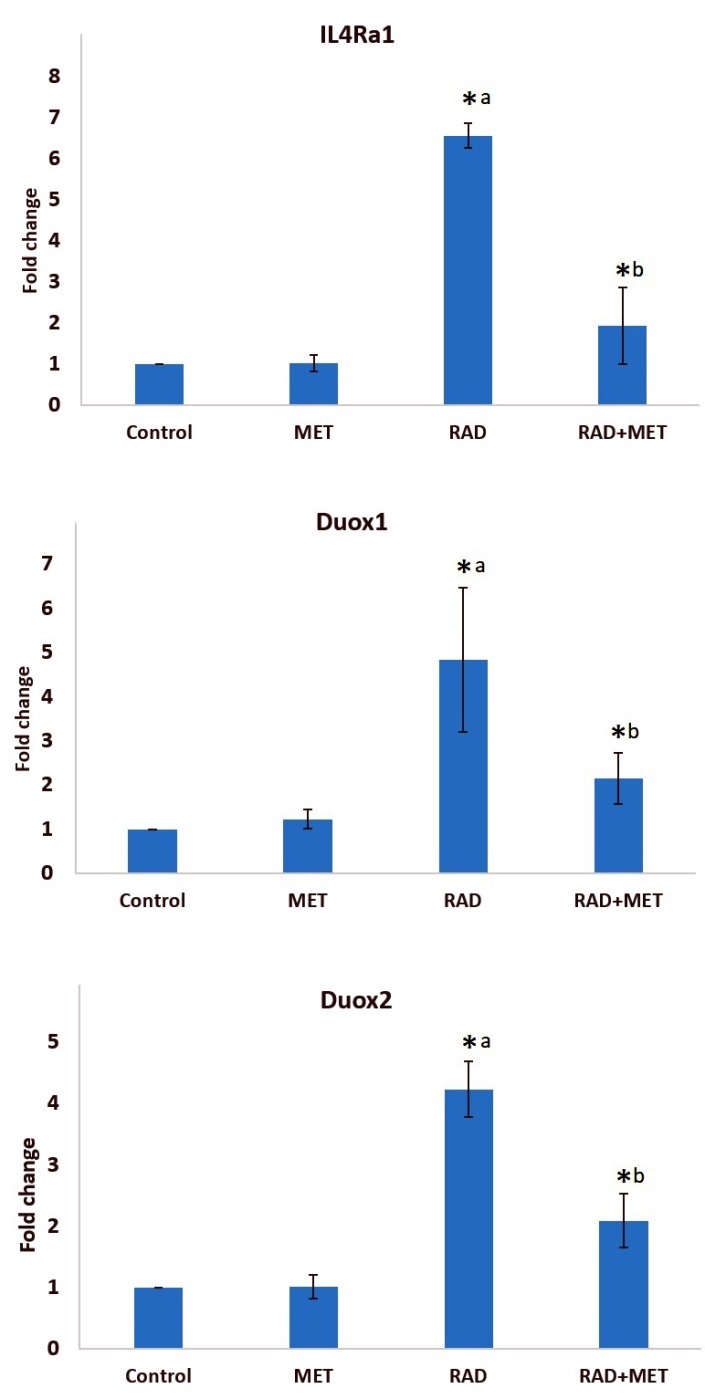
The expression of IL4Ra1, IL13Ra2 and DUOX2 in rat’s lung tissues following irradiation, treatment with metformin, and both, (t-test, *p <0.05 is considered a significant difference, a: significant compared to control group; b significant compared to radiation group). RAD=radiation; MET=metformin.

In this study, we aimed to evaluate the level of two important pro-fibrotic cytokines; IL-4 and IL-13, and their downstream genes, including IL4ra1, IL13Ra2, DUOX2 and DUOX1 in rat’s lung tissue following exposure to a high dose of radiation. We also examined the radioprotective effect of metformin on development of pneumonitis and fibrosis, infiltration of macrophages and lymphocytes, alveolar and vascular damage, as well as edema and collagen deposition. As shown in Figure 1, the expression of IL4ra1 was significantly increased following exposure to IR when compared to control group (6.55±0.30, p=0.001). However, in metformin treatment group, there was no significant change in the expression of this gene. Treatment with metformin could attenuate the expression of IL4ra1 significantly, compared with IR group (1.91±0.93, p <0.05). Unlike IL4ra1, there was no detectable expression of IL-13ra2 gene in all groups. The expression of DUOX1 increased following exposure to IR when compared to control group (4.83±1.63, p<0.05). Treatment with metformin could attenuate the expression of IL-4r compared to IR group (2.15±0.57, p <0.05). Results of DUOX2 gene expression showed a significant increase for the radiation group compared with control group (4.23±0.57, p ≤0.001). Moreover, its expression was significantly reduced in rats which were treated with metformin (2.09±0.44, p ≤0.001).

### ELISA

**Figure 2:**
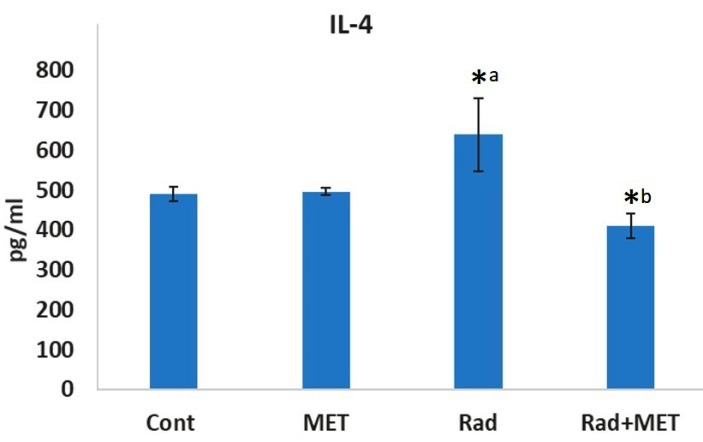
The levels of IL-4 in rat’s lung tissues following irradiation or treatment with metformin, or both. a: significant compared to control group; b significant compared to radiation group (ANOVA, Tukey's HSD, p<0.05). RAD=radiation; MET=metformin.

**Figure 3:**
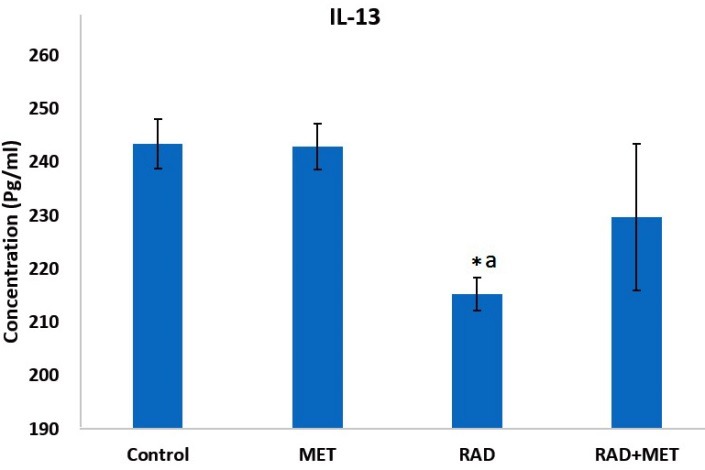
The levels of IL-13 in rat’s lung tissues following irradiation of rat’s chest area. a: significant compared to control group (ANOVA, Tukey's HSD, p<0.05). (ANOVA, Tukey's HSD, p<0.05). RAD=radiation; MET=metformin.

IL-4 and IL-13 are among important cytokines that induce ROS production. As earlier mentioned, IL4Ra1 and IL13Ra2 are the main receptors of these cytokines. As upregulation of these genes are involved in chronic oxidative stress and fibrosis, we hypothesized that the increased expression of these genes may be associated with pathological damages to the lung tissue. In addition, we evaluated the levels of IL-4 and IL-13 cytokines as well as pathological changes. Afterwards, we examined the protective effect of metformin on these changes. As shown in Figures 2 and 3, irradiation of lung tissue led to an increase in IL-4 level (640±43 pg/ml vs 413±30 pg/ml). However, treatment with metformin did not cause any change in the level of this cytokine. When rats were treated with metformin before irradiation, the level of IL-4 was suppressed compared to irradiation only group (339±36 pg/ml vs 640±43 pg/ml). In contrast to IL-4, the level of IL-13 was significantly reduced following exposure to IR (243±4 pg/ml vs 215±3 pg/ml), (ANOVA, Tukey's HSD, p=0.034). However, no changes were observed for treatment with metformin before and after irradiation compared with IR group (229±14 pg/ml).

### Histopathological analyses

**Figure 4:**
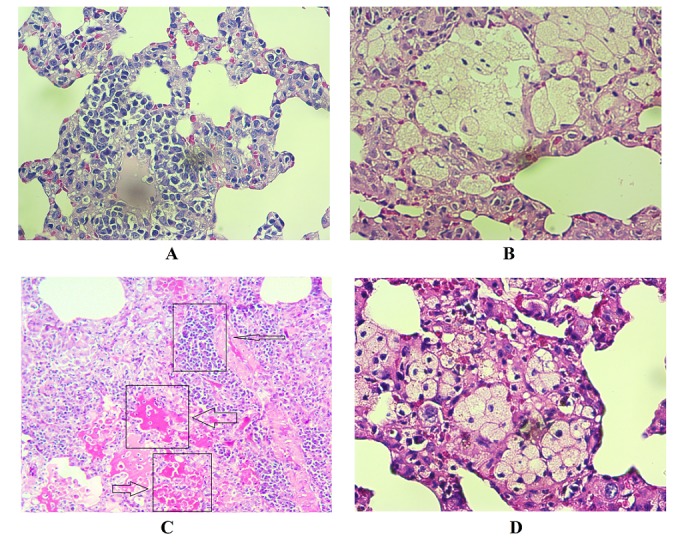
Histopathological effects of irradiation of rat’s lungs and protective effect of metformin. A: Normal; B: Radiation; C: Radiation + Metformin. Irradiation caused a mild interstitial infiltration of lymphoplasma, and significant elevation of foam cells. Treatment with metformin before and after irradiation caused suppression of all changes in histological parameters (H&E staining).

**Figure 5:**
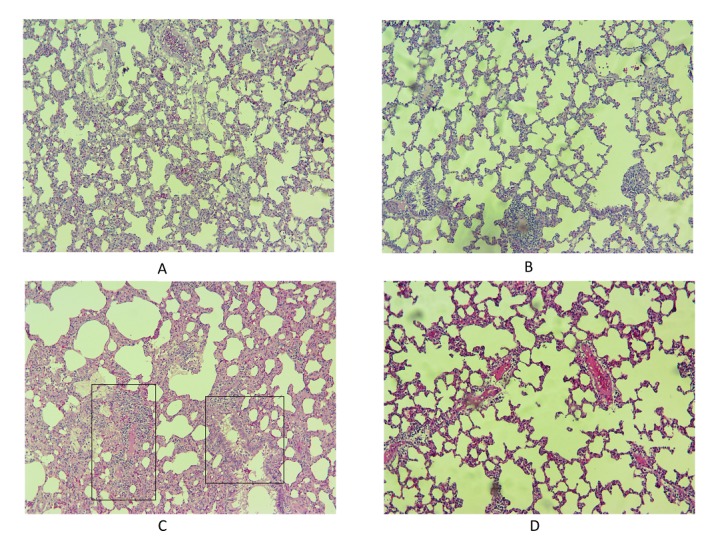
Histopathological effects of irradiation of rat’s lungs and protective effect of metformin. Radiation led to mild fibrosis, while metformin reversed it completely. (Massons trichrome staining)

As shown in Table 2, irradiation led to an increase in infiltration of macrophages and lymphocytes, thickening of alveolar and vascular endothelium as well as edema and thrombosis. Treatment with metformin led to attenuation of all mentioned factors. In addition, irradiation caused a mild fibrosis, which was suppressed by metformin (Figures 4 and 5). It is well known that macrophages and lymphocytes are able to release large numbers of cytokines that lead to appearance of inflammation and fibrosis. Macrophages and lymphocytes, through some cytokines induce ROS and NO production that have key roles in promotion of pneumonitis and collagen deposition.

**Table 2:** Results of histopathological evaluation of rat’s lung tissues, radiation group was compared to control group while radiation plus metformin group was compared to radiation group (a: significant compared to control group, ANOVA, Tukey's HSD, p<0.05).

	Control	Metformin treated	Radiation	Radiation+Metformin
Macrophage infiltration	1.00±00	1.00±00	3.66±0.57^a^	1.5±0.57^b^
Lymphocyte infiltration	1.75±0.50	1.00±00	4.00±00^a^	1.5±0.57^b^
Neutrophil infiltration	1.25±50	1.00±00	1.00±00	1.25±50
Alveolar thickness	1.00±00	1.00±00	2.66±0.57^a^	1.25±50^b^
Vascular thickness	1.00±00	1.00±00	2.00±00^a^	1.00±00^b^
Edema and thrombosis	1.00±00	1.00±00	2.00±00^a^	1.00±00^b^

Metformin is able to modulate cellular metabolism via inhibition of reduction/oxidation reactions and inflammation. A study by Sato et al. has shown that metformin through inhibition of TGF-β–NOX4 signaling pathway attenuate Smad phosphorylation and myofibroblast differentiation, leading to reduced lung fibrosis.^16^ Modulatory effects of metformin have been proposed for protection against radiation injury. It has also shown ability to reduce IR-induced cellular damage as reported in vitro and in vivo studies.^35,36^ Metformin has shown reduced cell death and micronucleus formation in human lymphocytes.^35^ An in vivo study by Xu et al. has shown that metformin suppresses long term upregulation of NOX4 in mice bone marrow stem cells following irradiation. This was associated with decreased ROS production, DNA damage and apoptosis. Also, metformin could stimulate the activity of antioxidant enzymes such as superoxide dismutase (SOD) and glutathione (GSH).^37^

Targeting IL-4 and IL-13 was proposed for mitigation of radiation injury in the lung.^38^ It has been shown that suppression of IL-4 can reduce late effects of radiation in this organ like fibrosis and macrophage accumulation.^34^ In this study, we showed that the expression of IL-4 and its downstream genes involved in lung injury after radiation therapy can be potently inhibited by metformin.

## Conclusion

In this study, we showed that irradiation of rat’s lung led to upregulation of IL-4–IL-4R1–DUOX2 pathway, associated with inflammation and infiltration of macrophages and lymphocytes. However, there was significant reduction in the level of IL-13. This could be an indication that upregulation of DUOX1 and DUOX2 by IL-4 is involved in lung injury following exposure to IR. Treatment with metformin could suppress pathological damages to the lung such as infiltration of macrophages and lymphocytes after exposure to a high dose of IR. This was associated with a reduction in IL-4 level as well as expression of IL4Ra1, DUOX1 and DUOX2 genes.

## Acknowledgments

The authors gratefully acknowledge the research council of Kermanshah University of Medical Sciences (grant number: 3005233) for financial support.

## Ethical Issues

This study was accordance to the principles outlined in “The Guide for The Care and Use of Laboratory Animals” prepared by Kermanshah University of Medical Sciences.

## Conflict of Interest

The authors declare that they have no conflict of interest.
